# Care Pathways and Initial Engagement in Early Psychosis Intervention Services Among Youths and Young Adults

**DOI:** 10.1001/jamanetworkopen.2023.33526

**Published:** 2023-09-13

**Authors:** Alexia Polillo, George Foussias, Wei Wang, Aristotle N. Voineskos, Jacqueline Veras, Nicole Davis-Faroque, Albert H.C. Wong, Nicole Kozloff

**Affiliations:** 1 Centre for Addiction and Mental Health, Toronto, Ontario, Canada; 2 Department of Psychiatry, University of Toronto, Toronto, Ontario, Canada; 3 Dalla Lana School of Public Health, University of Toronto, Toronto, Ontario, Canada

## Abstract

**Importance:**

Broad efforts to improve access to early psychosis intervention (EPI) services may not address health disparities in pathways to care and initial engagement in treatment.

**Objective:**

To understand factors associated with referral from acute hospital-based settings and initial engagement in EPI services.

**Design, Setting, and Participants:**

This retrospective cohort study used electronic medical record data from all patients aged 16 to 29 years who were referred to a large EPI program between January 2018 and December 2019. Statistical analysis was performed from March 2022 to February 2023.

**Exposures:**

Patients self-reported demographic information in a structured questionnaire. The main outcome for the first research question (referral source) was an exposure for the second research question (initial attendance).

**Main Outcomes and Measures:**

Rate of EPI referral from acute pathways compared with other referral sources, and rate of attendance at the consultation appointment.

**Results:**

The final study population included 999 unique patient referrals. At referral, patients were a mean (SD) age of 22.5 (3.5) years; 654 (65.5%) identified as male, 323 (32.3%) female, and 22 (2.2%) transgender, 2-spirit, nonbinary, do not know, or prefer not to answer; 199 (19.9%) identified as Asian, 176 (17.6%) Black, 384 (38.4%) White, and 167 (16.7%) other racial or ethnic groups, do not know, or prefer not to answer. Participants more likely to be referred to EPI services from inpatient units included those who were older (relative risk ratio [RRR], 1.10; 95% CI, 1.05-1.15) and those who identified as Black (RRR, 2.11; 95% CI, 1.38-3.22) or belonging to other minoritized racial or ethnic groups (RRR, 1.79; 95% CI, 1.14-2.79) compared with White participants. Older patients (RRR, 1.16; 95% CI, 1.11-1.22) and those who identified as Black (RRR, 1.67; 95% CI, 1.04-2.70) or belonging to other minoritized racial or ethnic groups (RRR, 2.11; 95% CI, 1.33-3.36) were more likely to be referred from the emergency department (ED) compared with White participants, whereas participants who identified as female (RRR, 0.51 95% CI, 0.34-.74) had a lower risk of ED referral compared with male participants. Being older (odds ratio [OR], 0.95; 95% CI, 0.90-1.00) and referred from the ED (OR, 0.40; 95% CI, 0.27-0.58) were associated with decreased odds of attendance at the consultation appointment.

**Conclusions and relevance:**

In this cohort study of patients referred to EPI services, disparities existed in referral pathways and initial engagement in services. Improving entry into EPI services may help facilitate a key step on the path to recovery among youths and young adults with psychosis.

## Key Points

**Question:**

What disparities exist in who is referred to early psychosis intervention (EPI) from acute hospital-based settings and initial engagement in services?

**Findings:**

In this cohort study of 999 patients referred to EPI, patients who were older and Black or from other minoritized racial and ethnic groups were more likely to be referred from the emergency department and inpatient units compared with White patients. Being older and referred from the emergency department were associated with nonattendance at the consultation appointment.

**Meaning:**

These findings suggest that disparities exist in care pathways and initial engagement in EPI services, and additional efforts are required to identify youths and young adults with psychosis, particularly those from structurally marginalized populations, and connect them with services.

## Teaser

This cohort study investigates factors associated with referral from acute hospital-based settings and initial engagement in early psychosis intervention (EPI) services among young adult patients.

## Introduction

Early psychosis intervention (EPI) is an approach that can encompass different models of care that aim to provide treatment early in the course of psychotic illness. The rationale for EPI has been strengthened by consistent findings that long duration of untreated psychosis (DUP) is associated with more severe symptoms, lesser likelihood of remission, and poor social functioning and global outcome.^1^ Despite the clear mandate for EPI programs to promote their services and minimize barriers to care, most youths with psychosis either never access these services, or access them far later than indicated.^2^ This is the case in Ontario, Canada, despite the availability of EPI services across the province.^3^

Pathways to EPI care, the process by which youths and their families access treatment, have been well studied with the goal of reducing treatment delays and DUP.^4,5^ This process involves identifying symptoms, initiating help-seeking, contact with mental health services, and EPI referral.^4,6^ During this time, youths and their families may encounter barriers that may delay help-seeking and access to treatment, including difficulty recognizing symptoms, lack of trust in clinicians, poor service coordination, and inappropriate referrals.^7^ A performance indicator of quality EPI care in fidelity assessments is the proportion of patients who have been hospitalized prior to admission, as this suggests local barriers accessing care, and international clinical practice guidelines reiterate the goal of avoiding inpatient admissions in the pathway to EPI services.^8,9^ Despite this, acute services act as 1 of the main entry points into care for youths with psychosis,^4,10^ with nearly half of all new psychotic disorders diagnosed in the emergency department (ED) or inpatient units.^11^ Although advancements have been made to improve access to EPI services, health disparities still exist in the early stages of accessing care, with evidence suggesting that Black patients are at higher risk of having acute pathways to care^12,13,14^; however, the use of self-reported demographic information is limited across studies.

Another clear gap in the literature is the lack of research on initial engagement in EPI services, even though it is a critical step in ongoing service engagement. Many studies have explored ongoing engagement in EPI services, identifying a lack of family involvement and problem substance use as robust predictors of EPI service disengagement.^15,16^ Other studies have examined EPI treatment entry. An Ontario study found that people indicated for EPI services were more likely to be assessed by an EPI program if they were younger and male, received their diagnosis from a psychiatrist and in an outpatient setting, and were less likely to live in areas of socioeconomic deprivation.^17^ The study was not able to examine data on race and ethnicity and the sample contained only a small proportion of first-generation migrants.^17^ Studies examining factors associated with initial engagement, defined as attendance at the first EPI appointment, are lacking, despite the fact that none of the benefits of EPI are possible if people with psychosis do not get in the door to receive care.

Our primary objective was to understand factors associated with acute referral to and initial engagement with EPI services, with a focus on self-reported health equity–related factors, using retrospective cohort data. EPI services in the context of this study reflect the NAVIGATE model of coordinated specialty care.^18^ Referral sources were defined as acute if the patient was referred from the ED or inpatient unit. Initial engagement was defined as attendance at the consultation appointment. We hypothesized that patients who identified as Black would be more likely to be referred from an acute setting compared with people from other racial and ethnic groups,^12,13,14^ and that acute referral would be associated with nonattendance at the consultation appointment.^17^

## Methods

### Study Design and Data Source

This retrospective cohort study was approved by the research ethics board at the Centre for Addiction Mental Health (CAMH). Informed consent was not required for this study, as it involved an analysis of retrospective electronic medical record (EMR) data that posed minimal risk. This study followed the Strengthening the Reporting of Observational Studies in Epidemiology (STROBE) reporting guideline for cohort studies.^19^ We obtained EMR data for all patients aged 16 to 29 years who were referred to the CAMH EPI program over a 2-year period from January 2018 to December 2019. Referral data were excluded from the analysis if the referral was cancelled for any reason (eg, the patient was found to not meet the service eligibility criteria, the referral was redirected to an EPI program closer to the patient’s home, or the patient was admitted to hospital or in police custody) and for patients previously enrolled in the CAMH EPI program. For patients with multiple referrals during the 2-year period, only data from the first referral were used in the primary analysis. Data cleaning was conducted manually, and missing data were manually extracted from patient EMRs when possible.

### Setting

The CAMH EPI program is the largest in Canada, serving downtown Toronto, the fourth largest city in North America (although some additional smaller programs have overlapping geographic coverage). The program provides consultation and 3 years of coordinated specialty care for people up to 29 years of age with affective, nonaffective, and substance-induced psychosis.^18^ Patients are referred to the EPI program through the ED, CAMH inpatient units, CAMH outpatient psychiatrists, or externally through primary care practitioners (PCP) or external inpatient and outpatient psychiatrists. The ED also houses a bridging clinic, where they may triage patients who are determined to be at lower acuity level during business hours; the bridging clinic is also used to provide short-term follow-up after an inpatient discharge. External referrals are received centrally by Access CAMH and triaged by nurses to appropriate services. Self-referral is also permitted, although only a small number are referred this way. Following the Ontario EPI Program Standards, the program aims to offer consultation appointments within 2 weeks.^3^

### Data Selection

This study had 2 outcomes of interest: (1) rate of EPI referral from acute pathways (ED, bridging clinic, or inpatient referrals) compared with other referral sources, and (2) rate of attendance at the EPI consultation appointment. Patients were coded as having not attended if they declined services, could not be reached to schedule their appointment, or did not attend their scheduled appointment. Demographic variables, including age, country of birth, and self-reported gender, racial and ethnic group (other racial and ethnic groups included Indigenous, Latin American, Middle Eastern, and other not specified), and sexual orientation were derived from CAMH’s self-report standardized health equity form that is routinely completed by patients around the time of their first appointment and during ED visits and inpatient admissions.^20^ Pathways to care variables were gathered from clinical documentation, including referral source and days to consult, calculated as the number of days from referral to consultation appointment. Recoded variables are outlined in eTable 1 in Supplement 1. Categories were aggregated following health equity best practices to protect participants’ privacy (due to small data cells of fewer than 5 people) and facilitate statistical comparisons.^20^ Responses were included in the analysis for patients who indicated “don’t know” or “prefer not to answer” on the structured health equity form as we considered them valid responses; items that had no response were categorized as missing.^20^

### Missing Data

Missing data ranged from 0% to 11.9% across covariates, with sexual orientation having the highest missing rate. No values were missing for the outcomes of interest. Given that missing data were limited to self-identified demographic items, imputation was not used.

### Statistical Analysis

First, descriptive statistics were used to calculate characteristics for the full sample of patients referred to EPI services and based on referral source. Second, we used multinomial logistic regression to model the risk of acute referral, while controlling for demographic factors including age, gender, racial and ethnic group, country of birth, and sexual orientation; calculating relative risk ratios (RRR) and 95% CIs. Third, we used binary logistic regression to model the odds of attendance at the consultation appointment, controlling for the same demographic factors as well as referral source and days to consultation appointment, calculating odds ratios (ORs) and 95% CIs. We performed univariable tests and then included variables statistically significant at an a priori level of *P* < .20 in a backward stepwise selection to determine the final adjusted models by removing variables with *P* ≥ .20. We conducted sensitivity analyses using mixed-effects binary logistic regression, modeling initial engagement when second and third referral data remained in the analysis. A 2-sided *P* < .05 was considered statistically significant. All analyses were conducted using Stata statistical software version 16.0 (StataCorp) from March 2022 to February 2023.^21^

## Results

**Figure 1.**
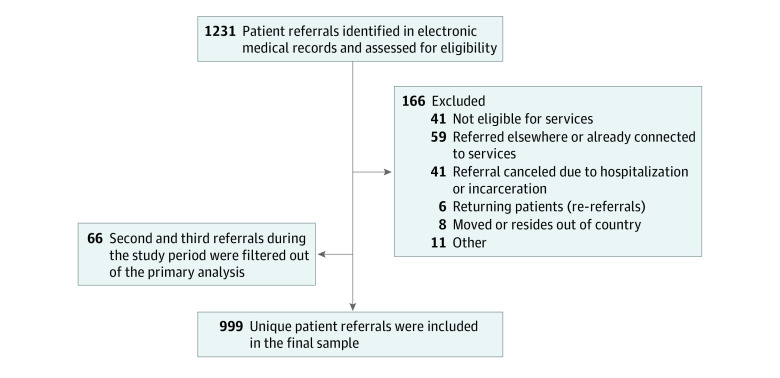
Study Sample of Patients Referred to Early Psychosis Intervention Services Between January 2018 and December 2019

**Figure 2.**
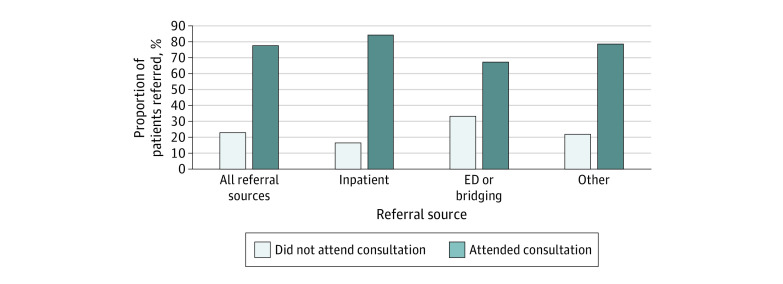
Attendance at Early Psychosis Intervention Program Consultation by Referral Source ED indicates emergency department.

The cohort included 1231 patient referrals received by the CAMH EPI program between January 2018 and December 2019, of which 999 referrals (mean [SD] age, 22.5 [3.5] years; 654 [65.5%] male, 323 [32.3%] female, and 22 [2.2%] transgender, 2-spirit, nonbinary, do not know, or prefer not to answer; 199 [19.9%] Asian, 176 [17.6%] Black, 384 [38.4%] White, and 167 [16.7%] other racial or ethnic groups, do not know, or prefer not to answer) met study eligibility after 66 second and third referrals were filtered out of the primary analysis (Figure 1 and Table 1). A total of 474 patients (47.4%) were referred from the ED, bridging clinic, or inpatient units compared with 525 (52.6%) who were referred from other referral sources. Figure 2 and eTable 2 in Supplement 1 show the proportion of patients who did and did not attend their consult from each referral source. Overall, 229 (22.9%) did not attend their consult; nonattendance was highest among those referred from the ED or bridging clinic (72 [33.2%]).

**Table 1.** Demographic Characteristics of Participants

Characteristic	Patients, No. (%)			
	All referral sources (N = 999)	Referral source		
		Inpatient (n = 257)	ED or bridging (n = 217)	Other (n = 525)^a^
Age, mean (SD), y	22.5 (3.5)	22.9 (3.1)	23.5 (3.3)	21.9 (3.6)
Age, median (IQR), y	22 (20-25)	23 (21-25)	24 (21-26)	21 (19-25)
Gender				
Male	654 (65.5)	160 (62.3)	162 (74.7)	332 (63.2)
Female	323 (32.3)	91 (35.4)	48 (22.1)	184 (35.1)
Transgender, 2-spirit, nonbinary, do not know, or prefer not to answer	22 (2.2)	6 (2.3)	7 (3.2)	9 (1.7)
Racial and ethnic group				
Asian	199 (19.9)	54 (21.0)	48 (22.1)	97 (18.5)
Black	176 (17.6)	62 (24.1)	40 (18.4)	74 (14.1)
White	384 (38.4)	87 (33.9)	74 (34.1)	223 (42.5)
Other racial and ethnic groups^b^	143 (14.3)	41 (16.0)	39 (18.0)	63 (12.0)
Do not know or prefer not to answer	24 (2.4)	8 (3.1)	10 (4.6)	6 (1.1)
Missing	73 (7.3)	5 (2.0)	6 (2.8)	62 (11.8)
Sexual orientation				
Heterosexual	667 (66.8)	184 (71.6)	159 (73.3)	324 (61.7)
LGBTQ2+	159 (15.9)	39 (15.2)	32 (14.8)	88 (16.8)
Do not know	54 (5.4)	21 (8.2)	13 (6.0)	20 (3.8)
Missing	119 (11.9)	13 (5.1)	13 (6.0)	93 (17.7)
Born in Canada				
Yes	606 (60.7)	165 (64.2)	121 (55.8)	320 (61.0)
No	294 (29.4)	74 (28.8)	84 (38.7)	136 (25.9)
Do not know or prefer not to answer	26 (2.6)	13 (5.1)	6 (2.8)	7 (1.3)
Missing	73 (7.3)	5 (2.0)	6 (2.8)	62 (11.8)
Days to consult, mean (SD)^c^	18.3 (13.9)	19.0 (12.4)	13.9 (11.0)	19.6 (15.2)
Days to consult, median (IQR)	15 (10-22)	16 (11-24)	12 (7-17)	16 (10-24)

Abbreviations: ED, emergency department; LGBTQ2+, lesbian, gay, bisexual, trans, queer (or sometimes questioning), and 2-spirited.

^a^ Other includes outpatient psychiatrists, primary care practitioners, or other external clinicians.

^b^ Other racial and ethnic groups included Indigenous, Latin American, Middle Eastern, and other not specified.

^c^ Includes data only for participants who booked a consultation appointment (n = 871).

### Assessment of Health Equity, Other Demographic Factors, and Referral Source

Table 2 displays the unadjusted and adjusted multinomial models comparing demographic characteristics of patients referred to EPI services from inpatient units, ED, bridging clinic, and other referral sources. In adjusted models, patients more likely to be referred to EPI services from inpatient units included those who were older (RRR, 1.10; 95% CI, 1.05-1.15) and those who identified as Black (RRR, 2.11; 95% CI, 1.38-3.22) or belonging to other racial and ethnic groups (including do not know or prefer not to answer) (RRR, 1.79; 95% CI, 1.14-2.79) compared with White participants. Older patients (RRR, 1.16; 95% CI, 1.11-1.22) and those who identified as Black (RRR, 1.67; 95% CI, 1.04-2.70) or belonging to other racial and ethnic groups (RRR, 2.11; 95% CI, 1.33-3.36) were also more likely to be referred from the ED or bridging clinic compared with White participants. Patients who identified as female (RRR, 0.51; 95% CI, 0.34-0.74) had a lower risk of ED referral compared with those who identified as male.

**Table 2.** Multinomial Logistic Regression Analysis of Health Equity Factors and Referral Source

Variable	Referral source			
	Inpatient vs other^a^		ED or bridging vs other^a^	
	RRR (95% CI)	*P* value	RRR (95% CI)	*P* value
**Unadjusted**				
Age	1.09 (1.04-1.13)	<.001	1.14 (1.09-1.20)	<.001
Gender				
Male	1 [Reference]	NA	1 [Reference]	NA
Female	1.03 (0.75-1.40)	.87	0.53 (0.37-0.77)	.001
Transgender, 2-spirit, nonbinary, do not know, or prefer not to answer	1.38 (0.48-3.95)	.55	1.59 (0.58-4.36)	.36
Racial and ethnic group				
Asian	1.43 (0.94-2.16)	.09	1.49 (0.97-2.30)	.07
Black	2.15 (1.41-3.26)	<.001	1.63 (1.02-2.60)	.04
White	1 [Reference]	NA	1 [Reference]	NA
Other racial and ethnic groups, do not know, or prefer not to answer^b^	1.82 (1.17-2.83)	.008	2.14 (1.36-3.36)	.001
Sexual orientation				
Heterosexual	1 [Reference]	NA	1 [Reference]	NA
LGBTQ2+ or do not know	0.98 (0.68-1.41)	.91	0.85 (0.57-1.26)	.42
Born in Canada				
Yes	1 [Reference]	NA	1 [Reference]	NA
No, do not know, or prefer not to answer	1.18 (0.85-1.63)	.32	1.66 (1.19-2.33)	.003
**Adjusted**				
Age	1.10 (1.05-1.15)	<.001	1.16 (1.11-1.22)	<.001
Gender				
Male	1 [Reference]	NA	1 [Reference]	NA
Female	0.92 (0.66-1.27)	.60	0.51 (0.34-0.74)	.001
Transgender, 2-spirit, nonbinary, do not know, or prefer not to answer^c^	NA	NA	NA	NA
Racial or ethnic group				
Asian	1.38 (0.90-2.10)	.14	1.52 (0.97-2.38)	.07
Black	2.11 (1.38-3.22)	.001	1.67 (1.04-2.70)	.04
White	1 [Reference]	NA	1 [Reference]	NA
Other racial and ethnic groups, do not know, or prefer not to answer^b^	1.79 (1.14-2.79)	.01	2.11 (1.33-3.36)	.002
Born in Canada				
Yes	1 [Reference]	NA	1 [Reference]	NA
No, do not know, or prefer not to answer^c^	NA	NA	NA	NA

Abbreviations: ED, emergency department; LGBTQ2+, lesbian, gay, bisexual, transgender, queer (or sometimes questioning), and 2-spirited; NA, not applicable; RRR, relative risk ratio.

^a^ Other is the reference group and includes referrals from outpatient psychiatrists, primary care practitioners, or other external clinicians.

^b^ Other racial and ethnic groups included Indigenous, Latin American, Middle Eastern, and other not specified.

^c^ Variables removed from the adjusted model though backward stepwise selection.

### Assessment of Health Equity, Demographic, and Pathways to Care Factors and Initial Engagement

Table 3 shows the unadjusted and adjusted binary logistic regression models for demographic and pathways to care factors and initial engagement. Following univariate tests and stepwise backward selection, being older (OR, 0.95, 95% CI, 0.90-1.00) and referred from the ED or bridging clinic (OR, 0.40, 95% CI, 0.27-0.58) were associated with decreased odds of attendance at the consultation appointment in the final adjusted model. Results were similar in sensitivity analyses, which used mixed effects logistic regression and included multiple referral data (eTable 3 in Supplement 1).

**Table 3.** Logistic Regression Analysis of Baseline Factors and Attendance at Early Psychosis Intervention Program Consultation

Variable	Unadjusted		Adjusted	
	OR (95% CI)	*P* value	OR (95% CI)	*P* value
Age	0.93 (0.89-0.97)	.001	0.95 (0.90-1.00)	.05
Gender				
Male	1 [Reference]	NA	1 [Reference]	NA
Female^a^	1.40 (1.01-1.95)	.05	NA	NA
Trans, 2-spirit, nonbinary, do not know, or prefer not to answer	0.70 (0.28-1.75)	.44	0.48 (0.18-1.27)	.14
Racial and ethnic group				
Asian	1.34 (0.83-2.16)	.23	1.43 (0.91-2.26)	.12
Black^a^	0.82 (0.53-1.29)	.39	NA	NA
White	1 [Reference]	NA	1 [Reference]	NA
Other racial and ethnic groups, do not know, or prefer not to answer^a^^,^^b^	0.72 (0.46-1.12)	.14	NA	NA
Sexual orientation				
Heterosexual	1 [Reference]	NA	1 [Reference]	NA
LGBTQ2+ or do not know^a^	0.77 (0.53-1.13)	.19	NA	NA
Born in Canada				
Yes	1 [Reference]	NA	1 [Reference]	NA
No, don’t know, or prefer not to answer^a^	1.14 (0.80-1.62)	.47	NA	NA
Referral source				
Other^c^	1 [Reference]	NA	1 [Reference]	NA
ED or bridging	0.56 (0.40-0.80)	.001	0.40 (0.27-0.58)	<.001
Inpatient^a^	1.44 (0.97-2.12)	.07	NA	NA
Days to consult	0.99 (0.98-1.01)	.42	NA	NA

Abbreviations: ED, emergency department; LGBTQ2+, lesbian, gay, bisexual, transgender, queer (or sometimes questioning), and 2-spirited; NA, not applicable; OR, odds ratio.

^a^ Variables removed from the adjusted model through backward stepwise selection.

^b^ Other racial and ethnic groups include Indigenous, Latin American, Middle Eastern, and other not specified.

^c^ Other is the reference group and includes referrals from outpatient psychiatrists, primary care practitioners, or other external practitioners.

## Discussion

Among 999 patients referred to EPI services between January 2018 and December 2019, approximately one-quarter did not attend the consultation appointment, indicating initial service engagement challenges. In contrast to past studies,^22,23^ we found that most patients were referred to EPI services through an outpatient psychiatrist, PCP, or other external referral source rather than acute settings. Those referred from inpatient units had the highest rate of attendance at the consultation appointment compared with other referral sources. Compared with younger and White patients, those who were older and identified as Black or other racial and ethnic groups were more likely to be referred from the ED or bridging clinic and inpatient units, and female patients were less likely to be referred from the ED or bridging clinic compared with male patients. Although equity-related factors were associated with EPI referral, they were not associated with initial engagement in EPI. Instead, being older and referred from the ED or bridging clinic were associated with nonattendance at the consultation appointment. These findings can help identify patients who may face barriers in the early stages of accessing care and inform improvements to initial engagement in EPI services.

We found that 22.9% of patients did not attend the consultation appointment. This was consistent with the attendance rate found in a study of missed first appointments among youth and adults with schizophrenia.^24^ This finding, along with evidence that approximately one-third of patients with early psychosis disengage from services prematurely,^15,16^ suggests that patients face engagement challenges throughout their service trajectories. Given that patients are often referred to EPI services at a critical stage in their illness (a time of heightened risk for suicidal behaviors and death) and that patients who receive EPI services have lower mortality rates and ED visits compared with those not receiving EPI services, improving timely access to care and initial engagement should be a priority for EPI programs and researchers.^25,26^

Whereas we had hypothesized that those patients referred from inpatient units would behave similarly to those from the ED and bridging clinic with respect to initial engagement, we found that, in fact, patients referred from inpatient units had the highest rate of attendance at the EPI consultation compared with other referral sources, and those referred from the ED and bridging clinic had the lowest. Hospitalizations may be an opportunity to initiate medication and stabilize symptoms, involve families in treatment planning, and connect patients with outpatient clinicians, all of which may facilitate attendance at the consultation. Although the ED has been found to be 1 of the main referral pathways to EPI services in past studies,^22,23^ we found that more than half of patients were referred to EPI services through an outpatient psychiatrist, PCP, or other external clinician, with most attending their consultation appointment. Shifting identification and appropriate referrals for people with psychosis from acute settings has been an aim of quality improvement initiatives and suggests that efforts to ensure young people have access to primary care,^11^ educate primary care and community-based clinicians about early psychosis,^3^ and implement coordinated and low-barrier referral systems may be helping connect patients to EPI services earlier in the course of illness.

We found that patients who identified as Black or other racial and ethnic groups were more likely to be referred from the ED or bridging clinic and inpatient units compared with White patients. Our findings add to the EPI evidence highlighting racial disparities in service access and use, with numerous studies documenting that Black patients are at higher risk of coercive referral and intervention, involuntary admissions, and emergency service use, relative to other racial and ethnic groups.^27^ In past research, these disparities have been explained by delays in help-seeking due to stigma or cultural and/or religious beliefs, mistrust in the medical system because of past negative experiences, racial bias leading to police involvement and coercive intervention, and other structural barriers including poverty and residential instability.^5,12,14,28,29^ These factors may also pose barriers to accessing care for other patients with minoritized race and ethnicity as they were also more likely to be referred from the ED or bridging clinic compared with White patients. Although access to EPI services may be improving in general, minoritized populations continue to face barriers accessing care. Systematically evaluating and monitoring outcomes through an equity lens can help identify gaps within EPI programs, leading to program improvements that make treatment more universally accessible.

Interestingly, equity-related factors did not emerge as associated with initial engagement in our study or later disengagement in other EPI studies,^16,30^ with problem substance use, medication nonadherence, and a lack of family support associated with disengagement later in EPI treatment.^16^ Although we were not able to test the association between these factors and initial engagement as these data were not available at the time of EPI referral, referral from the ED or bridging clinic was found to be associated with nonattendance at the initial appointment and may act as a proxy for acuity or decreased social support. More research is needed to identify factors associated with initial EPI engagement as it has been largely unexplored until now.

The association between ED or bridging clinic referral and nonattendance is a troubling but unsurprising finding, given that in a study of young people in Ontario presenting with a psychotic disorder to the ED for the first time, almost half received no outpatient mental health follow-up within 30 days of the ED visit.^31^ In that study, it was unknown if the lack of mental health follow-up was due to nonreferral or nonattendance; our study found that of patients referred to EPI services from the ED or bridging clinic, approximately one-third do not attend the initial appointment, suggesting an engagement rather than referral problem. Improving the transition from the ED to EPI services has the potential to decrease the DUP and subsequent ED visits,^26^ and improve symptoms and functioning by urgently connecting youths who present in crisis with EPI services.^32,33^

Similar to past research on EPI access and disengagement,^17,23^ we found that older age was associated with nonattendance at the initial appointment. This finding may be explained by patients with older age being less likely to have family members involved in their care as they enter adulthood and gain more autonomy, which is consistent with previous studies of EPI disengagement that found an association between family involvement and shorter delays in help-seeking.^16,34^ Another potential explanation is that older patients, particularly those referred from the ED or bridging clinic, may have a longer DUP and more severe symptoms leading to nonattendance. Delays in treatment have been associated with poorer clinical and functional outcomes,^35^ as well as a heightened risk of disengagement from EPI services, albeit this evidence is mixed.^16,36^ This further supports the need for targeted approaches to engage patients in EPI care, including those in later stages of early adulthood.

### Limitations

This study has limitations. We were not able to distinguish between referrals from external PCPs, outpatient psychiatrists, and external inpatient units; however, we suspect the number of external inpatient referrals to be small based on a medical record review study that found that there were no external inpatient referrals among 225 patients enrolled in the CAMH EPI program from July 2018 to May 2019. Despite having a large sample size, some categories had to be combined (due to having fewer than 5 people in them) to protect the anonymity of patients, limiting our ability to detect within-group differences across all variables. Other factors, including substance use and symptom severity, may better explain ED or bridging clinic referral and attendance at the consultation appointment but these data were not available at the time of referral to services. Some of the gender response options in the hospital’s health equity questionnaire were categories more accurately associated with sex (male, female) rather than gender (men, women).

## Conclusions

In this cohort study of patients referred to EPI services, disparities existed in pathways to EPI care, and referrals from the ED or bridging clinic were at an elevated risk of not connecting to outpatient services. Future research should examine interventions that target the critical period between EPI referral and the consultation appointment, with the goal of building a therapeutic relationship and engaging patients in services as soon as possible, identifying potential contributors to and solutions for the differential pathways to accessing services in minoritized racial and ethnic populations, and exploring the experiences of those who declined EPI services to facilitate the development of multilevel interventions. Initial contact with EPI services provides the foundation for ongoing service engagement. Thus, improving the transition from EPI referral to EPI services may help facilitate a key step on the path to recovery among youths and young adults with psychosis.

## Notes

Supplement 1. **eTable 1.** Recoded Variables **eTable 2.** Rates of Attendance at Consultation Appointment by Referral Source **eTable 3.** Sensitivity Analysis of Factors Associated with Attendance at Initial Appointment Including Data of Participants with >1 Referral

Supplement 2.
Data Sharing Statement
