# Supplementary material for: Care Pathways and Initial Engagement in Early Psychosis Intervention Services Among Youths and Young Adults
Source: JAMA Netw Open. 2023 Sep 13;6(9):e2333526. doi: 10.1001/jamanetworkopen.2023.33526 (PMC10500372; doi:10.1001/jamanetworkopen.2023.33526)
Supplement: Supplement 1. — eTable 1. Recoded Variables eTable 2. Rates of Attendance at Consultation Appointment by Referral Source eTable 3. Sensitivity Analysis of Factors Associated with Attendance at Initial Appointment Including Data of Participants with >1 Referral [file jamanetwopen-e2333526-s001.pdf]

Supplemental Online Content

Polillo A, Foussias G, Wang W, et al. Care pathways and initial engagement in early psychosis intervention services among youths and young adults. *JAMA Netw Open.* 2023;6(9):e2333526. doi:10.1001/jamanetworkopen.2023.33526

eTable 1. Recoded Variables

eTable 2. Rates of Attendance at Consultation Appointment by Referral Source

eTable 3. Sensitivity Analysis of Factors Associated with Attendance at Initial Appointment Including Data of Participants with >1 Referral

This supplemental material has been provided by the authors to give readers additional information about their work.

© 2023 Polillo A et al. *JAMA Network Open.*

eTable 1. Recoded Variables

Variable	Recoding
Gender	Male, female, or other including trans, two-spirit, non-binary, don't know, and prefer not to answer
Racial/ethnic group	Asian, Black, White, or other racial/ethnic groups, don't know, and prefer not to answer
Sexual orientation	Heterosexual or LGBTQ2+ and don't know
Country of birth	Yes or no, don't know and prefer not to answer
Referral source	Inpatient, ED/bridging clinic, or outpatient psychiatrists, PCPs, and other external providers
Attendance at consultation appointment	Attended or did not attend

Abbreviations: LGBTQ2+, lesbian, gay, bisexual, trans, queer (or sometimes questioning), and two-spirited; ED, emergency department; PCP, primary care provider.

© 2023 Polillo A et al. *JAMA Network Open.*

eTable 2. Rates of Attendance at Consultation Appointment by Referral Source

		Referral source		
**Outcome of referral**	**All referral sources (n = 999)**	**Inpatient (n = 257)**	**ED/Bridging (n = 217)**	**Other[a] (n = 525)**
Attended consult	770 (22.9)	215 (83.7)	145 (66.8)	410 (78.1)
Did not attend consult[b]	229 (77.1)	42 (16.3)	72 (33.2)	115 (21.9)

[a] Other includes outpatient psychiatrists, PCPs, or other external providers.

[b] Nonattendance at consult includes those who declined services, could not be reached for booking, or booked and did not attend.

© 2023 Polillo A et al. *JAMA Network Open.*

eTable 3. Sensitivity Analysis of Factors Associated with Attendance at Initial Appointment Including Data of Participants with >1 Referral

	**Unadjusted**		**Adjusted**	
**Variable**	**OR (95% CI)**	***P***	**OR (95% CI)**	***P***
Age	.92 (0.88-0.97)	.002	0.96 (0.91-1.01)	.12
Gender	-	-	-	-
Male	1 [Reference]	NA	1 [Reference]	NA
Female	1.53 (1.05-2.24)	.03	1.29 (0.87-1.91)	.20
Trans, two-spirit, non-binary, don't know, or prefer not to answer	0.59 (0.21-1.68)	.32	0.54 (0.20-1.41)	.21
Racial/ethnic group	-	-	-	-
Asian	1.38 (0.86-2.22)	.18	1.25 (0.77-2.04)	.37
Black	0.79 (0.51-1.23)	.30	0.76 (0.48-1.20)	.24
White	1 [Reference]	NA	1 [Reference]	NA
Other racial/ethnic groups, don't know, or prefer not to answer[a]	0.71 (0.45-1.10)	.12	0.81 (0.51-1.28)	.36
Sexual orientation	-	-	-	-
Heterosexual	1 [Reference]	NA	1 [Reference]	NA
LGBTQ2+ or don't know	0.75 (0.51-1.08)	.13	0.75 (0.50-1.13)	.17
Born in Canada	-	-	-	-
Yes	1 [Reference]	NA	-	-
No, don't know, or prefer not to answer	1.13 (0.79-1.62)	.50	-	-
Referral source	-	-	-	-
Other[b]	1 [Reference]	NA	1 [Reference]	NA
ED/bridging	.53 (0.35-0.82)	.004	0.39 (0.26-0.59)	<.001
Inpatient	1.53 (0.98-2.39)	.06	0.86 (0.56-1.32)	.49
Days to consult	0.10 (0.98-1.01)	.64	-	-

Abbreviations: OR, odd ratio; CI, confidence interval; NA, not applicable; LGBTQ2+, lesbian, gay, bisexual, trans, queer (or sometimes questioning), and two-spirited; ED, emergency department; PCP, primary care provider.

[a] Other racial/ethnic groups includes Indigenous, Latin American, Middle Eastern, and other not specified.

[b] Other includes referrals from outpatient psychiatrists. PCPs, or other external providers.

© 2023 Polillo A et al. *JAMA Network Open.*
