# Supplementary material for: Care Pathways and Initial Engagement in Early Psychosis Intervention Services Among Youths and Young Adults
Source: JAMA Netw Open. 2023 Sep 13;6(9):e2333526. doi: 10.1001/jamanetworkopen.2023.33526 (PMC10500372; doi:10.1001/jamanetworkopen.2023.33526)
Supplement: Supplement 2. — Data Sharing Statement [file jamanetwopen-e2333526-s002.pdf]

# Data Sharing Statement

Polillo. Care Pathways and Initial Engagement in Early Psychosis Intervention Services Among Youths and Young Adults. *JAMA Netw Open*. Published September 13, 2023. doi:10.1001/jamanetworkopen.2023.33526

**Data**
**Data available:** No

**Additional Information**
**Explanation for why data not available:** The data that support the findings of this study are available from the corresponding author, NK, upon reasonable request to protect the privacy and confidentiality of participants.
